# Meniscus-Derived Matrix Scaffolds Promote the Integrative Repair of Meniscal Defects

**DOI:** 10.1038/s41598-019-44855-3

**Published:** 2019-06-18

**Authors:** Jacob C. Ruprecht, Taylor D. Waanders, Christopher R. Rowland, James F. Nishimuta, Katherine A. Glass, Jennifer Stencel, Louis E. DeFrate, Farshid Guilak, J. Brice Weinberg, Amy L. McNulty

**Affiliations:** 1 Department of Biomedical Engineering, Duke University, Durham, NC USA; 2 Department of Mechanical Engineering and Materials Science, Duke University, Durham, NC USA; 3 Department of Orthopaedic Surgery, Duke University School of Medicine, Durham, NC USA; 4 Department of Medicine, Duke University School of Medicine, Durham, NC USA; 5 Department of Pathology, Duke University, Durham, NC USA; 6 Department of Orthopaedic Surgery, Washington University, St. Louis, MO USA; 7 Shriners Hospitals for Children – St. Louis, St. Louis MO, USA; 8 VA Medical Center, Durham, NC USA

**Keywords:** Cartilage, Skeleton

## Abstract

Meniscal tears have a poor healing capacity, and damage to the meniscus is associated with significant pain, disability, and progressive degenerative changes in the knee joint that lead to osteoarthritis. Therefore, strategies to promote meniscus repair and improve meniscus function are needed. The objective of this study was to generate porcine meniscus-derived matrix (MDM) scaffolds and test their effectiveness in promoting meniscus repair via migration of endogenous meniscus cells from the surrounding meniscus or exogenously seeded human bone marrow-derived mesenchymal stem cells (MSCs). Both endogenous meniscal cells and MSCs infiltrated the MDM scaffolds. In the absence of exogenous cells, the 8% MDM scaffolds promoted the integrative repair of an *in vitro* meniscal defect. Dehydrothermal crosslinking and concentration of the MDM influenced the biochemical content and shear strength of repair, demonstrating that the MDM can be tailored to promote tissue repair. These findings indicate that native meniscus cells can enhance meniscus healing if a scaffold is provided that promotes cellular infiltration and tissue growth. The high affinity of cells for the MDM and the ability to remodel the scaffold reveals the potential of MDM to integrate with native meniscal tissue to promote long-term repair without necessarily requiring exogenous cells.

## Introduction

The menisci are C-shaped fibrocartilaginous tissues found between the femoral condyles and tibial plateau in the knee. The menisci serve to distribute loads, provide a low friction surface for joint loading and movement, and are critical for maintaining the normal biomechanical function of the knee^1–4^. Meniscal tears, particularly those in the avascular inner zone, have a poor healing capacity. Damage to the meniscus is associated with significant pain and disability, as well as degenerative changes in the knee joint that ultimately lead to osteoarthritis (OA)^5–11^. Approximately two-thirds of patients with meniscal tears develop radiographic knee OA within 5–15 years of injury^12,13^. Current orthopaedic practice aims to preserve meniscal integrity and restore function through a variety of different methods. These techniques include fixation devices, abrasion, trephination, biocompatible meniscus scaffolds (such as the collagen meniscus implant)^14,15^, and allograft transplantation. In the United States, 850,000 meniscal surgeries are performed annually, and there are nearly twice as many performed worldwide^16^. Despite favorable short-term results, none of these treatments have yielded long-term regeneration of functional meniscal tissue or prevented OA development^17^. Therefore, strategies to promote meniscus repair and induce long-term regeneration of functional meniscus tissue are needed.

In this regard, tissue engineering approaches that use biomaterial scaffolds in combination with endogenous or exogenous cells provide a novel approach for biological repair of meniscal injuries^17,18^. In particular, extracellular matrix (ECM)-derived scaffolds have been shown to promote tissue healing and repair for a variety of orthopaedic applications. For example, demineralized bone matrix has been used to enhance bone regeneration and repair^19^, and a cartilage-derived matrix has been shown to promote neocartilage formation^20^. Additionally, these tissue-derived matrices direct stem cell differentiation towards a phenotype similar to the tissue of origin^21–26^. There are several advantages of tissue-derived matrices over other scaffold types for tissue engineering applications. In particular, tissue-derived scaffolds contain native components of the ECM and promote cell infiltration and remodeling^27,28^. Tissue-derived scaffolds may also contain natural growth factors localized to the ECM^27^ and thus may minimize the need for exogenous growth factor addition. In addition, these scaffolds may promote cellular infiltration and tissue repair by enabling cells to interact with their native ECM components.

Several prior studies have generated meniscus tissue-derived scaffolds for either tissue repair or replacement. Whole meniscus tissue has been decellularized to generate allograft scaffolds for meniscus tissue replacement^29–34^. However, these scaffolds maintain the dense ECM that limits cellular migration into the meniscus^35^, which is necessary for biological repair of meniscus tissue. Other studies have used physical disruption^36^, chemical treatments, enzymatic treatments, or a combination thereof to generate scaffolds^6,37–39^ or hydrogel additives^23–26,40,41^ from meniscus tissue that could potentially be utilized for meniscus repair.

The objective of this study was to use only physical disruption of porcine meniscus tissue to generate minimally processed meniscus-derived matrix (MDM) scaffolds and test their effectiveness in promoting integrative meniscus repair. Meniscus repair and integration with meniscus tissue was evaluated using different densities of MDM with and without scaffold crosslinking. The scaffolds were filled by endogenous meniscus cells migrating from the injured meniscus tissue or seeded with exogenous bone marrow-derived mesenchymal stem cells (MSCs). Additionally, exogenous growth factors were not used, in order to evaluate the ability of endogenous cells or MSCs seeded on the MDM scaffolds to promote meniscus repair. In this study, we provide evidence that the MDM scaffold is a promising tool for integrative meniscus repair.

## Results

### Generation of MDM scaffolds for meniscus tissue repair

**Figure 1:**
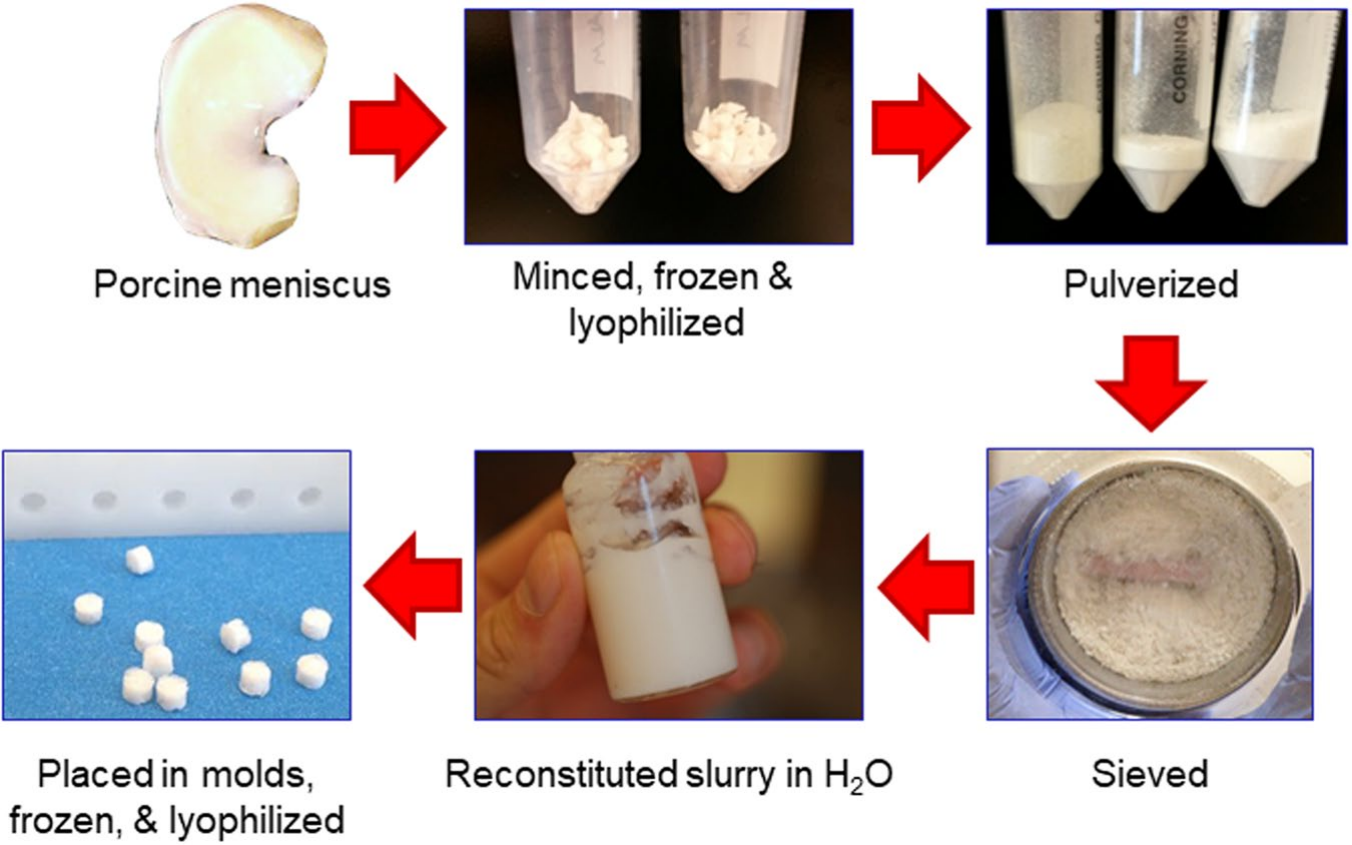
Meniscus-derived matrix (MDM) was prepared from porcine medial menisci that were minced, frozen, and lyophilized. Next, the tissue was pulverized and sieved, and then resuspended at 4% or 8% in water. The MDM slurry was pipetted into 3 mm diameter molds, which were frozen and lyophilized to generate scaffolds.

Porcine medial menisci were harvested, minced, frozen, lyophilized, and pulverized in a freezer mill (Fig. 1). The powder was run through a 500 μm sieve to remove large particles and rehydrated to 4% or 8% concentration by weight with distilled water. The resulting slurry was homogenized on ice^42^ and pipetted into a mold with cylindrical holes. Next, the scaffolds were frozen, lyophilized, and removed from the mold. Half of the scaffolds at each concentration were dehydrothermally crosslinked (X) by heating at 120 °C for 24 hr in a dry oven^22,43^.

**Figure 2:**
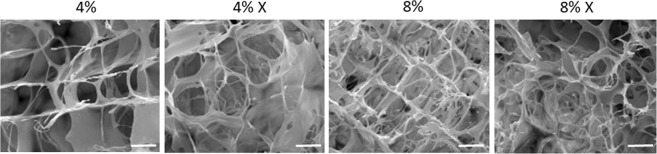
Scanning electron microscope images of the MDM scaffolds showing the porosity and structure of the 4%, 4% crosslinked (X), 8%, and 8% X scaffolds. Scale bar is 50 μm.

Scanning electron microscope (SEM) imaging was performed on MDM scaffolds to visualize the physical structure of the scaffolds (Fig. 2). The pore diameters were as follows (mean ± SEM): 81 ± 4 μm for the 4% MDM, 77 ± 4 μm for the 4% X MDM, 53 ± 3 μm for the 8% MDM, and 53 ± 2 μm for the 8% X MDM scaffolds. On average the 4% MDM scaffolds had significantly larger pore diameters than the 8% scaffolds (p < 0.000001). However, there was no effect of crosslinking on pore diameter. In all scaffolds, pores were larger than cell diameters (10–12 μm)^44^ and appear to be interconnected to facilitate cellular penetration into the scaffolds.

### Imaging of MDM scaffolds cultured with or without meniscus tissue

**Figure 3:**
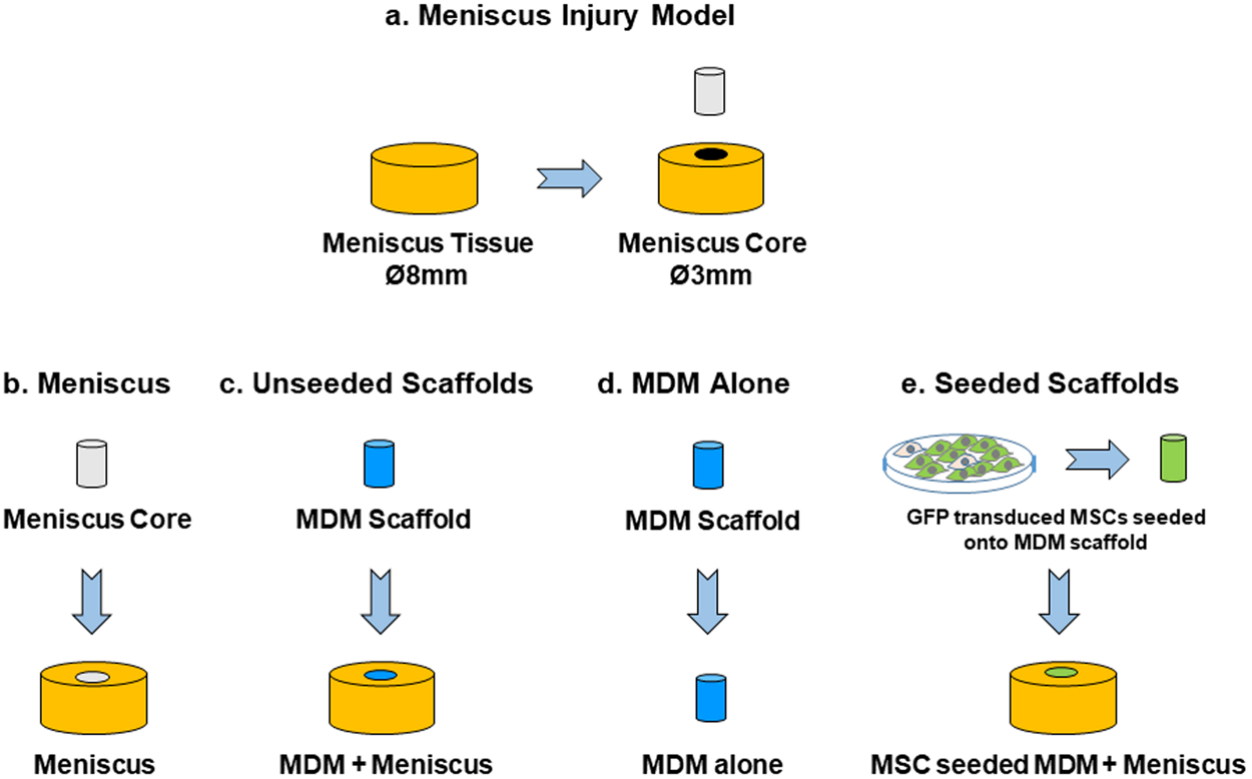
Schematic showing the experimental model and groups used in these experiments. (**a**) For the meniscus injury model, 8 mm diameter explants were isolated from porcine meniscus tissue. A 3 mm biopsy was removed from the center of the explant. (**b**) For meniscus tissue controls, the 3 mm inner core of meniscus tissue was reinserted into the meniscus explant (Meniscus). (**c**) For experiments with unseeded scaffolds, the inner core was filled with an acellular MDM scaffold (MDM + Meniscus). (**d**) MDM scaffolds were also cultured alone to assess the biochemical composition of the scaffold alone (MDM alone). (**e**) In order to generate seeded scaffolds, MSCs were transduced with enhanced green fluorescent protein (eGFP) and seeded on the MDM scaffolds. These MSC seeded MDM scaffolds were used in the inner core of the meniscus tissue (MSC seeded MDM + Meniscus) to assess the effects of MSCs on the MDM scaffold and meniscus repair.

An *in vitro* model of meniscal injury was created using porcine medial meniscus tissue cut to 8 mm diameter and 2 mm thick explants with a 3 mm diameter core removed from each explant to simulate a full-thickness defect^45–50^ (Fig. 3a). For control samples (Meniscus) the inner core was immediately reinserted into the defect (Fig. 3b), while in the experimental groups (Unseeded Scaffolds) the defect was filled with a 4%, 4% crosslinked (4% X), 8%, or 8% crosslinked (8% X) MDM scaffold (MDM + Meniscus) (Fig. 3c). Scaffolds were cultured without tissue (MDM alone) to allow baseline characterization of the scaffold composition (Fig. 3d). The samples were cultured in meniscus growth media and harvested after 7 or 28 days in culture for fluorescence imaging, and at 28 days for biochemical analyses, mechanical testing, and histological analysis.

**Figure 4:**
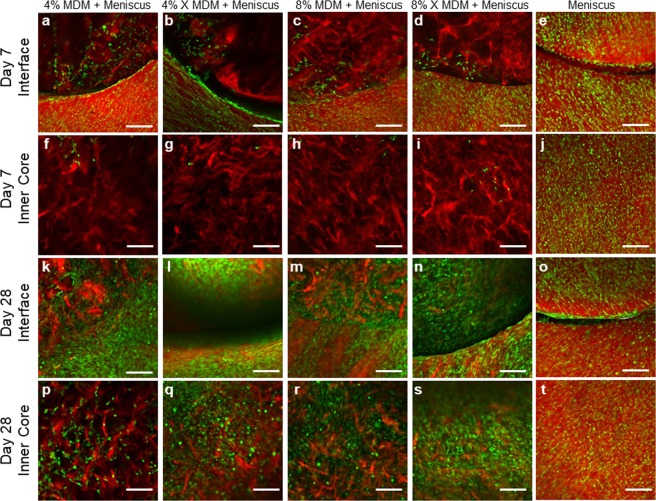
Fluorescent images of the unseeded MDM scaffolds in meniscus tissue (MDM + Meniscus) and meniscus tissue controls (Meniscus) at day 7 (**a**–**j**) and day 28 (**k**–**t**). Initially, the meniscus tissue contains abundant cells throughout (**e**,**j**) and by day 28 cells are bridging the interface between the inner core and outer ring of meniscus tissue (**o**). For the samples containing the MDM scaffold inner cores (Meniscus + MDM), the meniscus outer ring contains abundant cells (**a**–**d**), and these cells are starting to populate the MDM scaffold at day 7 (**f**–**i**). By day 28, the meniscus cells fully bridge the interface (**k**–**n**) and have filled the MDM scaffold inner cores (**p**–**s**). All meniscus cells are stained green (calcein AM) and the matrix is stained red (Alexa fluor 633 NHS ester). Scale bar is 200 μm.

Fluorescence imaging at day 7 revealed that meniscus cells migrated from the outer meniscus tissue and infiltrated the periphery of the unseeded scaffolds (Fig. 4a–d). However, very few meniscus cells migrated into the core of the scaffold (Fig. 4f–i). Conversely, there were abundant meniscus cells throughout the outer ring (Fig. 4e) and inner core (Fig. 4j) in the meniscus tissue control. At day 28, the meniscal cells had completely bridged the meniscus-scaffold interface (Fig. 4k–n) and infiltrated throughout the scaffold inner core (Fig. 4p–s). The control meniscus tissue was still filled with meniscus cells at day 28 (Fig. 4t), but fewer cells had bridged the interface between the meniscus inner core and outer ring (Fig. 4o) as compared to the MDM + Meniscus samples.

### Biochemical evaluation of MDM scaffolds cultured with or without meniscus tissue

**Figure 5:**
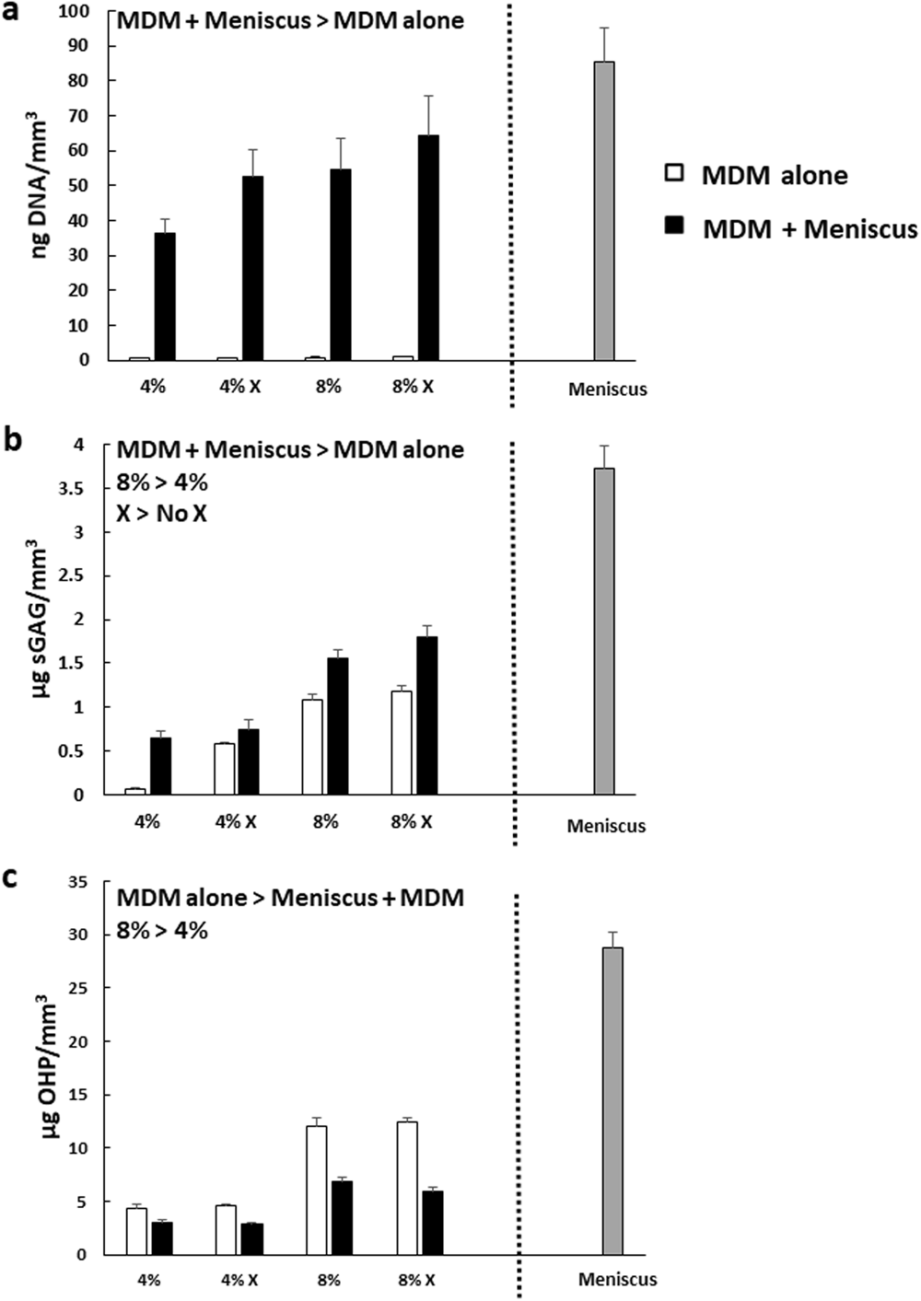
Meniscus tissue surrounding the MDM scaffolds (MDM + Meniscus) leads to increased DNA content (**a**) and sulfated glycosaminoglycan (sGAG) content (**b**) but decreased collagen (OHP) content (**c**) in the scaffolds. Data is expressed as the mean + SEM for the MDM alone (white bars) and the MDM + Meniscus (black bars). For reference the mean + SEM values for control meniscus tissue is indicated by the gray bar.

The scaffolds from the MDM + Meniscus groups had significantly higher DNA content relative to the MDM alone, indicating that native meniscal cells infiltrated the scaffold (Fig. 5a, p < 0.000001). There was no significant effect of scaffold composition (4% vs 8%) or crosslinking on the DNA content. Most notably, the DNA content of the MDM + Meniscus scaffolds was similar to the DNA content of the native meniscus tissue after 28 days.

The sulfated glycosaminoglycan (sGAG) content of the scaffolds was greater when cultured with meniscus tissue, as compared to MDM alone (Fig. 5b, p < 0.000001). In addition, the 8% scaffolds had higher sGAG content than the 4% scaffolds (p < 0.01) and crosslinked scaffolds contained more sGAG than the uncrosslinked scaffolds (p < 0.005). The native meniscus tissue contained only 2–3-fold higher sGAG content than the MDM scaffolds.

The collagen content, measured as hydroxyproline (OHP), of the scaffolds was lower in the MDM + Meniscus scaffolds than the MDM alone (Fig. 5c, p < 0.01). There was no significant effect of crosslinking on collagen content. However, the 8% scaffolds had higher collagen content than the 4% scaffolds (p < 0.01). In addition, there was an interactive effect of MDM concentration and tissue revealing the following significant differences in collagen content between groups: 4% MDM + Meniscus group < 4% MDM alone <8% MDM + Meniscus <8% MDM alone (p < 0.01). The native meniscus tissue contained only approximately 3-fold higher collagen content than the MDM scaffolds.

### Imaging of MSC seeded MDM scaffolds cultured with meniscus tissue

In order to assess the potential use of MSCs in the MDM scaffolds to promote meniscus repair, MSCs were transduced with lentivirus to constitutively overexpress enhanced green fluorescent protein (eGFP) and then seeded onto 4%, 4% X, 8%, or 8% X MDM scaffolds (Fig. 3e). The expression of eGFP allowed visualization of the MSCs on the MDM scaffolds and in the meniscus tissue. After creating meniscus injuries using our *in vitro* model (Fig. 3a), the defect was immediately filled with the meniscus tissue inner core for control samples (Meniscus) (Fig. 3b) or a scaffold seeded with MSCs for the experimental groups (Fig. 3e) (MSC seeded MDM + Meniscus). Samples were cultured in meniscus growth media and harvested after 7 or 28 days for fluorescence imaging and at 28 days for biochemical analyses, mechanical testing, and histological analysis.

**Figure 6:**
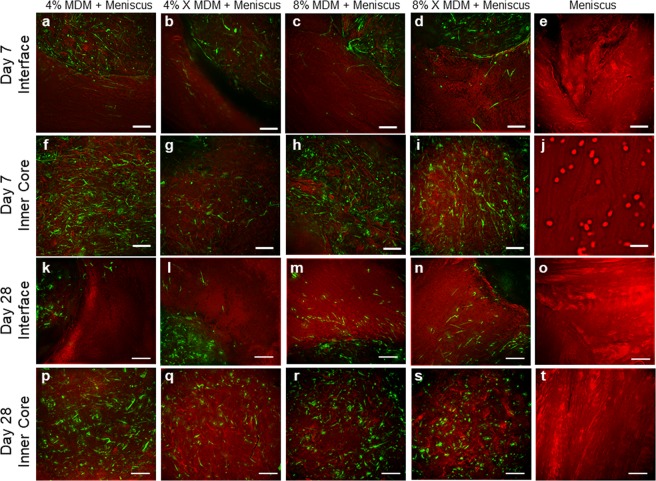
Fluorescent images of the MSC seeded MDM scaffolds in meniscus tissue (MSC seeded MDM + Meniscus) and meniscus tissue controls (Meniscus) at day 7 (**a**–**j**) and day 28 (**k**–**t**). Initially, the enhanced green fluorescent protein-expressing MSCs (eGFP-MSC) are localized to the MDM scaffold inner core (**f**–**i**). By day 28 the eGFP-MSCs have bridged the interface between the scaffold and outer ring of meniscus tissue and infiltrated into the meniscus tissue outer ring (**k**–**n**), particularly in the 8% groups (**m**,**n**). There are no eGFP-expressing cells in the meniscus tissue control (**e**,**j**,**o**,**t**). EGFP-MSCs are green and the matrix is stained red (Alexa fluor 633 NHS ester). Scale bar is 100 μm.

Fluorescence imaging of seeded scaffolds at day 7 showed eGFP-expressing MSCs throughout the scaffold inner cores (Fig. 6f–i). In addition, some MSCs had migrated from the scaffold into the surrounding meniscus tissue (Fig. 6a–d). At day 28, the MSCs were still evident throughout the MDM scaffold (Fig. 6p–s) and had infiltrated further into the meniscus outer ring tissue (Fig. 6k–n). On the other hand, no GFP expressing cells were visible in the meniscus tissue control at either day 7 (Fig. 6e,j) or day 28 (Fig. 6o,t).

### Biochemical evaluation of MSC seeded MDM scaffolds cultured with meniscus tissue

**Figure 7:**
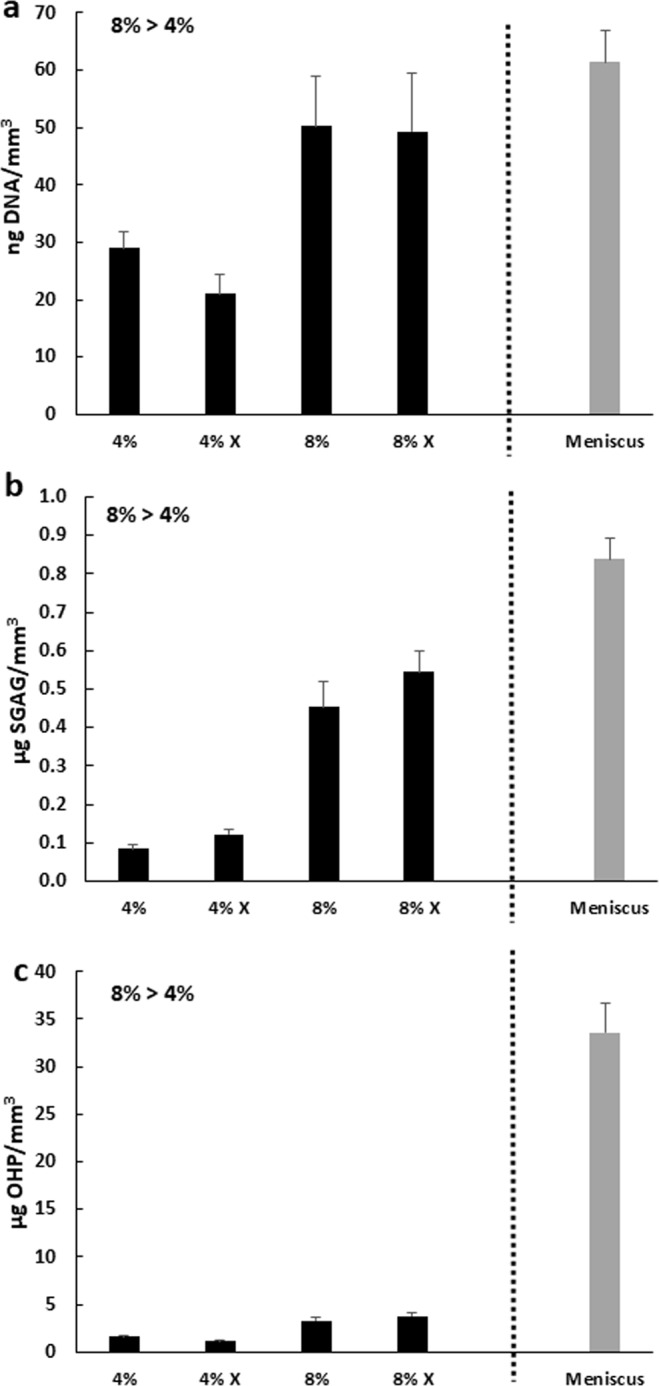
The 8% MDM MSC seeded scaffolds have higher DNA content (**a**), sulfated glycosaminoglycan (sGAG) content (**b**), and collagen (OHP) content (**c**) as compared to the 4% MDM scaffolds. Data is expressed as the mean + SEM for MSC seeded MDM + Meniscus (black bars). For reference the mean + SEM values for control meniscus tissue is indicated by the gray bar.

The 8% MSC seeded scaffolds had significantly higher DNA content relative to the 4% MSC seeded scaffolds (Fig. 7a, p < 0.001), indicating that the more concentrated scaffolds could support more cells. However, there was no significant effect of crosslinking on the DNA content. Most notably, the DNA content of the 8% seeded scaffolds after 28 days of culture surrounded by meniscus tissue was similar to the DNA content of the native meniscus tissue.

The 8% MSC seeded scaffolds had higher sGAG (Fig. 7b, p < 0.000001) and collagen content (Fig. 7c, p < 0.000001) than the 4% MSC seeded scaffolds. However, there was no significant effect of crosslinking on the sGAG or collagen content of the seeded scaffolds. The native meniscus tissue control contained 2-fold higher sGAG content and approximately 10-fold higher collagen content than the 8% seeded scaffolds.

### Mechanical testing to assess the shear strength of repair of the MDM scaffolds with meniscus tissue

**Figure 8:**
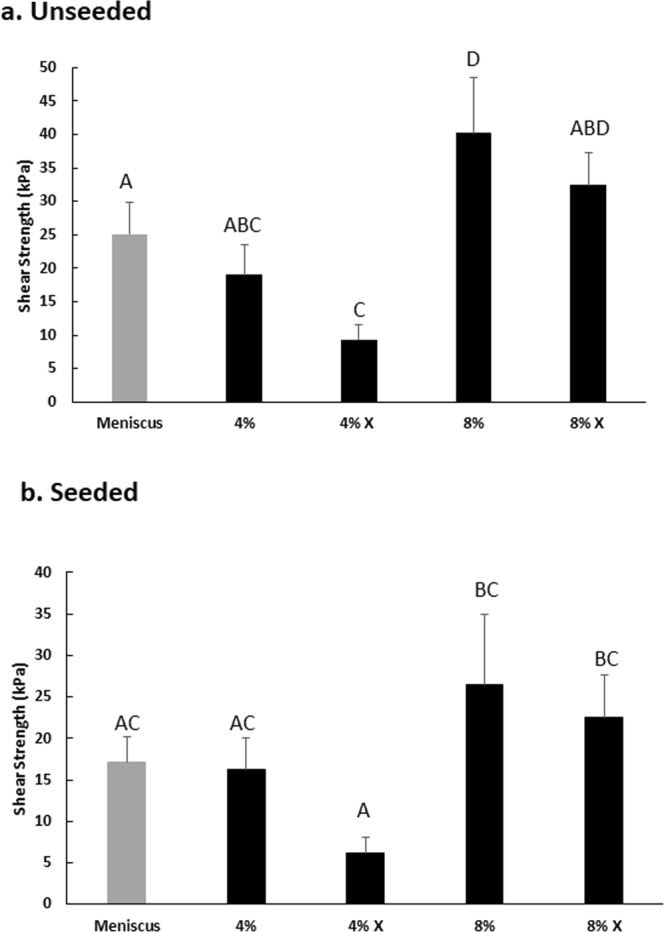
(**a**) The unseeded 8% MDM scaffolds have a higher integrative shear strength of repair with the meniscus tissue as compared to the control meniscus tissue. (**b**) The 8% and 8% X MSC seeded MDM scaffolds have improved repair strength over the 4% X MSC seeded MDM scaffolds. Data is expressed as the mean + SEM. Groups not sharing the same letter have p-values ≤ 0.05.

Push-out testing was used to assess the integrative shear strength of repair^45,46,48–51^ between the various inner cores and the outer ring of meniscus tissue after 28 days of culture. For the unseeded MDM scaffolds, the 8% scaffolds had a 160% higher shear strength of repair than the control meniscus tissue (Fig. 8a, p = 0.05) and also repaired better than the 4% and 4% X scaffolds (p < 0.01). The 4% X scaffolds also had significantly reduced shear strength of repair, as compared to the control meniscus tissue (p < 0.05). On the other hand, the 8% X scaffolds trended towards increased repair strength compared to the control meniscus tissue, but only had improved repair strength compared to the 4% X scaffolds (p < 0.01).

For the MSC seeded MDM scaffolds, both the 8% and 8% X scaffolds had a higher shear strength of repair compared to the 4% X scaffolds (Fig. 8b, p < 0.05). While on average the 8% seeded scaffold groups had a higher shear strength of repair than the control meniscus tissue, no significant difference was detected (p = 0.2).

### Histological analysis of MDM scaffolds cultured with meniscus tissue

**Figure 9:**
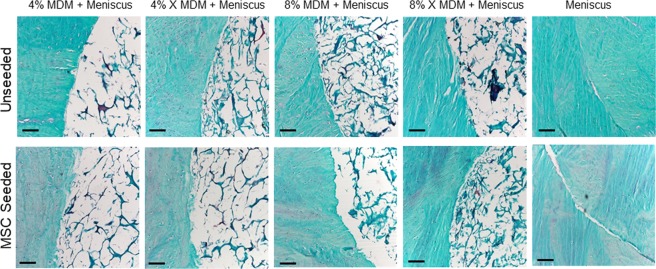
Histological staining of the MDM scaffolds cultured with meniscus tissue reveals the tissue structure and composition at the interface between the meniscus tissue and MDM scaffolds. Sections were stained with Safranin-O (red: proteoglycans), fast green (blue: collagen), and hematoxylin (black: nuclei). Scale bar is 100 μm.

After 28 days in culture, safranin O, fast green, and hematoxylin staining was performed to visualize the tissue structure at the interface between the meniscus tissue and MDM scaffolds. Histological analysis revealed that the MDM scaffolds had integrated with the surrounding meniscus tissue (Fig. 9). The ECM was composed predominantly of a collagenase matrix; however proteoglycan staining was apparent as well. Overall, more ECM was apparent in the samples containing 8% MDM scaffolds. All MDM scaffolds were more porous than the meniscus tissue control.

## Discussion

The MDM scaffold is a promising tool to enhance meniscus repair. The unseeded 8% MDM scaffolds improved the integrative repair strength of an *in vitro* meniscal defect as compared to a native meniscal tissue implant. In the absence of exogenous cells, crosslinking and concentration of the MDM influenced the biochemical content and shear strength of repair, revealing that the properties of the scaffolds can be modulated to promote tissue repair. Meniscal cells migrated from the surrounding meniscus tissue into the scaffolds and produced sGAGs, but decreased the collagen content of the scaffolds. Likewise, exogenous MSCs also showed high infiltration into the scaffolds, and higher MDM concentrations promoted the growth and retention of more cells. In the MSC seeded scaffolds, MDM concentration but not crosslinking influenced the biochemical and mechanical properties. The high affinity of cells for this scaffold, combined with their ability to remodel the scaffold is promising for the potential of MDM to integrate seamlessly into the native meniscal tissue to promote repair.

A major barrier to integrative meniscus repair is the dense ECM that prevents endogenous cells from readily migrating through the tissue to the tear site. Previous work has shown that the extracellular matrix of the adult meniscus sterically hinders cell mobility^35,52^. Therefore, our approach was to generate porous scaffolds that allowed migration of native meniscus cells into the scaffolds to form reparative tissue. The quantity of native meniscus cells on the unseeded scaffolds was not influenced by either the MDM concentration or crosslinking, although there was a trend towards more cells on the higher percentage scaffolds. In addition to being readily populated by native cells, the scaffolds were also able to support the growth and expansion of MSCs. In this case, there were more cells on the more concentrated MDM scaffolds. Remarkably, in only 28 days the number of either native meniscus cells or MSCs was quite similar to the number of cells found in the meniscus tissue control, indicating the high affinity of these cells for the MDM scaffold.

The MDM scaffold processing procedures resulted in retention of only approximately 1% of the DNA content of the meniscus control. On average, the MDM scaffolds contained 25 ng DNA per mg dry weight, which is lower than the recommended criteria for decellularization (<50 ng DNA/mg dry weight)^53^. Previous work has shown that *in vivo* there was no significant difference in M2:M1 macrophage ratios in response to implanted collagen scaffolds supplemented with 50 ng DNA or equivalent cellular amounts of mitochondria or cell membranes, as compared to collagen scaffolds alone^54^. However, *in vivo* experiments would be necessary to rule out immunoreactivity to our MDM scaffolds. Nonetheless, our reduction in DNA content is equivalent to or better than that seen with chemical and enzymatic decellularization protocols for meniscus, which range from 10 to 100-fold decreases in DNA content^24,25,37–41^. Furthermore, many of these prior decellularization protocols also reduced proteoglycan content^25,40,41,55^. Even after 28 days in culture, in this study the 8% scaffolds alone contained approximately 50% of the sGAG content of the meniscus tissue control, highlighting the ability of our scaffold preparation protocol to retain proteoglycans. As expected, there was greater sGAG and collagen content in the 8% scaffolds than the 4% scaffolds. However, the crosslinking of the scaffolds allowed for the retention of even more proteoglycans. This result is consistent with prior experiments using cartilage-derived matrix^22^ and with prior work demonstrating that dehydrothermal crosslinking results in covalent crosslinks between collagen and chondroitin sulfate^56^. Interestingly, there was greater sGAG content in the MDM + Meniscus samples than the MDM cultured alone, indicating that the infiltrating meniscus cells produced sGAGs that were retained in the scaffold.

The MDM + Meniscus samples had lower collagen content than the MDM alone, indicating a degradation of the collagen matrix in the presence of the meniscus cells. Dehydrothermal treated collagen matrices are more sensitive to trypsin digestion, which suggests that the crosslinking may partially denature the collagen matrix^57^. However, the decrease in collagen content in our study was independent of crosslinking, suggesting that the meniscus cells are remodeling the MDM scaffold and have not yet synthesized new collagen matrix. Future studies may explore the addition of physiologic mechanical stimulation to enhance collagen production and alignment^58^.

The enhanced integrative repair strength using the MDM shows the potential of the scaffold to improve tissue healing. There was increased repair beyond that seen with the meniscus control in the unseeded 8% MDM scaffolds. This work underscores the fact that native meniscus cells can enhance meniscus healing if provided with a favorable scaffold to promote cellular infiltration. Therefore, the use of exogenous cells may not be necessary to improve integrative meniscus tissue repair. However, one advantage of cell-based therapies is that they can be tailored to allow modulation of the injury microenvironment. For example, MSCs expressing inducible IL-1 receptor antagonist (IL-1ra) seeded on 3D woven polycaprolactone or cartilage-derived scaffolds have been used to modulate the pro-inflammatory environment^59,60^ that is present following joint injury and in OA. Therefore, future work could investigate whether genetically modified cells can further improve meniscus healing beyond that which is possible with native meniscus cells.

In these experiments, exogenous growth factors were not added to the culture media. We relied on the growth factors and matrix proteins that were retained in the MDM scaffolds to stimulate matrix production. However, prior studies have shown that chondrogenic media containing TGF-β3 was necessary for chondrogenic differentiation of MSCs in methacrylated gelatin hydrogels with meniscus matrix extract^25^ or decellularized meniscus scaffolds^37^. Connective tissue growth factor or TGF-β3 has also been shown to promote more robust chondrogenesis of infrapatellar fat pad stem cells as compared to control cultures in alginate functionalized with meniscus ECM^26^. While the combination of TGF-β3 and insulin-like growth factor (IGF)-1 was necessary to induce chondrogenesis of synovial fluid MSCs in a meniscus-derived matrix^36^. Previously, we found that TGF-β1 enhances meniscus cell accumulation in the interface of an injured meniscus explant and improves integrative shear strength of repair^47^. Several other studies have utilized chondrogenic media to promote matrix gene expression, ECM production, or both by MSCs in scaffolds containing meniscus-derived matrix^23–25^. Without the use of exogenous growth factors, our MDM scaffold was able to promote cellular migration into the scaffold, sGAG production, and improved integrative repair. The addition of soluble exogenous growth factors, tethering growth factors to the MDM, or introducing viral delivery systems to the MDM scaffold might further enhance tissue production and improve fibrochondrogenesis and integrative repair; however, this would increase the difficulty of translating this native MDM scaffold into the clinic for meniscus repair applications.

Early work on meniscus matrix scaffolds focused on the use of whole menisci for meniscus replacement^29–34^, which is necessary for degenerative joints and complex meniscus tears. However, our MDM scaffold can be used to reintegrate two pieces of meniscus tissue for radial, peripheral, longitudinal, or horizontal flap tears. As evidenced by the porous nature of our MDM scaffolds even after culture, the MDM scaffold is not mechanically robust enough to functionally replace the whole meniscus tissue. However, pre-fabricated MDM scaffolds could be used to bridge small gaps in a meniscus lesion to promote cellular infiltration and integrative repair. Alternatively, the MDM could be injected into lesions as a slurry to function as a biological glue. Further studies in meniscus-injured animal models will be necessary to translate the MDM scaffold to clinical use.

In this study, we provide evidence that the MDM scaffold is a promising tool for integrative meniscus repair. The scaffold provides a favorable environment for cellular infiltration and remodeling, and can enhance integrative repair. Thus, it has the potential to integrate seamlessly into meniscal lesions and promote repair without the need for an exogenous cell source or external growth factors. The high affinity of native meniscal cells to infiltrate the acellular MDM scaffolds, suggests that MDM is a favorable scaffold material to recruit native cells and promote long-term meniscal repair and homeostasis.

## Methods

### Scaffold fabrication

Medial menisci were harvested from the knees of skeletally mature 2–3 year old female pigs obtained from a local abattoir (n = 33), minced into ≤5 mm pieces, frozen overnight at −80 °C, and then lyophilized (FreeZone 2.5 L, Labconco, Kansas City, MO) for 24 h (Fig. 1). The lyophilized meniscus pieces were pulverized (5 min pre-cool, 5 cycles of 1 min at 5 Hz and 2 min cool) in a 6770 freezer mill (SPEX SamplePrep, Metuchen, NJ). The powder was sieved (500 µm) and pooled into a superlot that was used for further experiments.

MDM powder was rehydrated to either 4% or 8% weight fraction by mixing 0.4 g or 0.8 g MDM powder with distilled water to achieve 10 g total mass. After resuspension, the MDM slurry was homogenized (PRO260, PRO Scientific Inc., Oxford, CT) at 30,000 rpm for 2 cycles of 2 min run, 2 min cool on ice^42^. This homogenized slurry was pipetted into a delrin mold to completely fill holes of 1.8 mm-2 mm deep x 3 mm diameter and leave no trapped air bubbles. A silicone lid was placed over the delrin mold containing MDM slurry and was frozen at −80 °C overnight.

The next day the silicon cover was removed from the mold, and the delrin base containing the frozen slurry was returned to −80 °C for 30 min to ensure the scaffolds were completely frozen. The delrin mold with scaffolds was lyophilized for 24 h and the scaffolds were removed. Half of the scaffolds at each concentration were dehydrothermally crosslinked (X) by heating at 120 °C for 24 h in a dry oven^22^. All scaffolds were ethylene oxide sterilized in scintillation vials.

### SEM imaging of scaffolds

The MDM scaffolds were sputter-coated (Desk IV, Denton Vacuum, Moorestown, NJ) with gold at 18 mA for 600 s, resulting in a 20 nm thick deposition. After coating, samples were scanned (FEI XL30 ESEM, Hillsboro, OR) at an accelerating voltage of 30 kV. Images were taken at 250x magnification. To characterize pore size, maximum pore diameters were measured from SEM images using Image J (NIH) (n = 20/group).

### Meniscus explant harvest and injury model

Medial menisci were harvested from the knees of skeletally mature female pigs. 8 mm diameter punches were taken along the centerline of the tissue and then cut to a uniform thickness of 2 mm. A 3 mm diameter inner core was removed from the explant to simulate a full-thickness defect^45–51^ (Fig. 3a). For control samples (Meniscus), the inner core was immediately returned to the defect (Fig. 3b). Tissue explants were then transferred to 12 well plates (Corning Life Sciences), which had previously been coated with 500 μL of 2% agarose (Bio-Rad, Hercules, CA) to prevent cell attachment. Explants were incubated for 1 hour in wash media containing DMEM high glucose (DMEM-HG; Invitrogen, Carlsbad, CA) supplemented with 10% penicillin/streptomycin/fungizone (Invitrogen). Next the explants were removed from the wash media and the defect was filled with a 4%, 4% X, 8%, or 8% X MDM scaffold for the experimental groups (MDM + Meniscus) (Fig. 3c). These scaffolds were either wetted with DMEM-HG (Invitrogen) immediately prior to placement in the defect (Fig. 3c) or seeded with eGFP-expressing MSCs (Fig. 3e) as described below. The explants with MDM cores were then cultured in 4 mL meniscus growth medium consisting of DMEM-HG supplemented with 10% FBS (AVH79983, HyClone), 1% penicillin-streptomycin (Invitrogen), 1% HEPES buffer (Invitrogen), 1% non-essential amino acids (Invitrogen), and 37.5 μg/mL ascorbic acid 2-phosphate (Sigma-Aldrich, St. Louis, MO) at 37 °C/5% CO_2_ with media changes every 3 days. Unseeded MDM scaffolds (MDM alone) were also cultured without tissue (Fig. 3d). Samples were harvested after 7 or 28 days for fluorescent imaging and at day 28 for mechanical testing, biochemical analyses, and histology.

### Lentivirus production

EGFP was previously cloned into a lentiviral vector ﻿with the constitutive human EF-1α promoter (Addgene plasmid 12250)^59,61^. Lentivirus was produced in HEK293T/17 ﻿(ATCC CRL-11268, Manassas, VA), concentrated ∼75-fold using Amicon Ultra 100 kDa MWCO filters (Millipore, Cork, Ireland), and titered as described previously^59^.

### MSC transduction and scaffold seeding

Excess bone marrow aspirate was obtained from three de-identified adult bone marrow transplant donors at Duke University School of Medicine, in an IRB-exempt waste tissue protocol. Human MSCs were isolated, expanded, and pooled into a superlot as described previously^42,60^. MSCs at passage 3 were plated at 1 × 10^6^ cells/T-225 flask (Corning). The next day, cells were transduced with lentivirus vectors (2.48 × 10^7^ infectious particles/mL) supplemented with 4 μg/mL polybrene. After 20 hr, MSC expansion media containing 2 ng/mL bFGF was added to the cells. Media was refreshed every 2 days. On day 6, cells were trypsinized, counted, and frozen in liquid nitrogen to ensure they would be at the same passage during all subsequent experiments.

GFP-expressing MSCs were thawed and expanded. Passage 4 cells were trypsinized and resuspended in MSC expansion medium at a density of 7.8 × 10^6^ cells/mL. MDM scaffolds (4%, 4% X, 8%, and 8% X) were placed in a silicone seeding mold with holes 3 mm in diameter x 2 mm deep. Then 8 μL of cell suspension was pipetted onto the top of each scaffold. The seeding mold was placed into a vacuum chamber for 45 seconds to improve cell infiltration^62^. Scaffolds were flipped and 8 μL of cell suspension was pipetted onto the other side of the scaffolds and a vacuum was pulled for another 45 seconds to encourage cellular infiltration throughout the entire volume of the scaffold. The scaffolds were placed in individual wells of 24 well low attachment plates (Corning) for 30 min to allow cellular attachment. Then 1 mL of supplemented MSC expansion media was added to each well. The cells were expanded on the MDM scaffolds for 6 days, with media replaced every 48 hr. After 6 days of cell expansion, the seeded scaffolds were placed into the outer ring of the meniscal explant injury model described above (Fig. 3e).

### Fluorescent imaging of constructs

For the unseeded MDM scaffold experiments, confocal microscopy (LSM 510, Zeiss, Thornwood, NY, USA) was used to visualize the interface between the inner core and outer ring of meniscus tissue, and the central portion of the various inner cores during culture. Meniscal repair model explants were harvested after 7 and 28 days in culture for imaging to visualize live cells (calcein AM, Invitrogen) and matrix (Alexa fluor 633 NHS ester, Invitrogen). For MSC seeded experiments, widefield fluorescent images (IX83, Olympus) were taken of eGFP-labeled MSCs and Alexa fluor 633 NHS ester stained matrix at days 7 and 28.

### Mechanical testing for the shear strength of repair

After 28 days of culture, explants were harvested for mechanical testing using a push-out test (n ≥ 9/group)^45,46,48–51^. Each sample was loaded onto a dish, which contained a 4 mm hole in the center, and placed in the loading frame (ELF 3200, EnduraTec, Minnetonka, MN). A 2 mm piston was connected to a load cell (Honeywell, Morris Plains, NJ) and centered directly above the inner core. A 0.5 g tare load was applied for 10 seconds, and then the piston was lowered to a final displacement of 4 mm at a rate of −0.083 mm/sec. Images taken of each construct were imported into Image J software to measure sample thickness and diameter. MATLAB (Mathworks, Natick, MA) was used to create force-displacement curves and shear strength was calculated as the peak force divided by the area of the repair interface.

### Biochemical analyses of MDM constructs

After mechanical testing, each MDM inner core was digested in 500 μL of papain (125 μg/mL papain (Sigma), 100 mM phosphate buffer, 10 mM cysteine, and 10 mM EDTA, pH 6.3) at 65 °C overnight^22,63,64^ for biochemical analyses (n ≥ 9/group). MDM scaffolds that were cultured alone were subjected to the same digest conditions (n = 6/group). The picogreen assay (Invitrogen) was used to measure DNA content. The sGAG content was measured using the dimethylmethylene blue assay^65^, and a bovine chondroitin sulfate standard (Sigma-Aldrich). Collagen content was assessed through the hydroxyproline assay^66^, which utilizes alkaline hydrolysis to cleave collagen into hydroxyproline, followed by reaction with chloramine-T and 4-(Dimethyl-amino)benzaldehyde^63,64^. Biochemical data was corrected for the volume of the scaffold.

### Histological analysis of constructs

After 28 days in culture, samples were fixed in formalin overnight at 4 °C, dehydrated, and paraffin embedded. Samples were cut into 8 μm sections and then stained with Harris’ hematoxylin (Poly Scientific), 0.02% aqueous fast green (Electron Microscopy Sciences), and 0.1% aqueous Safranin-O (Sigma-Aldrich) in order to visualize cell nuclei, collagen, and proteoglycans respectively.

### Statistical analyses

Statistical analyses were performed using Statistica (Tibco). All data were normally distributed. For the biochemical outcomes, factorial ANOVA and Fisher LSD post hoc tests were performed to determine significant group differences (α = 0.05), as well as the interactive effects of concentration, crosslinking, and tissue. In order to allow statistical comparison to the meniscus control group, one-way ANOVA and Fisher LSD post hoc tests were performed for the shear strength data.

## Data Availability

All data generated or analyzed during this study are included in this published article.
